# A framework to estimate a long-term power shortage risk following large-scale earthquake and tsunami disasters

**DOI:** 10.1371/journal.pone.0283686

**Published:** 2023-03-27

**Authors:** Yoshio Kajitani, Daisuke Takabatake, Ayumi Yuyama, Tomomi Ishikawa, Wolfgang Kröger

**Affiliations:** 1 Faculty of Engineering and Design, Department of Engineering and Design, Kagawa University, Takamatsu, Kagawa, Japan; 2 Sustainable System Research Laboratory, Central Research Institute of Electric Power Industry, Abiko, Chiba, Japan; 3 Professor Emeritus, ETH Zurich, Zurich, Switzerland; National Institute of Technology Silchar, India, INDIA

## Abstract

While power shortages during and after a natural disaster cause severe impacts on response and recovery activities, related modeling and data collection efforts have been limited. In particular, no methodology exists to analyze long-term power shortages such as those that occurred during the Great East Japan Earthquake. To visualize a risk of supply shortage during a disaster and assist the coherent recovery of supply and demand systems, this study proposes an integrated damage and recovery estimation framework including the power generator, trunk distribution systems (over 154 kV), and power demand system. This framework is unique because it thoroughly investigates the vulnerability and resilience characteristics of power systems as well as businesses as primary power consumers observed in past disasters in Japan. These characteristics are essentially modeled by statistical functions, and a simple power supply–demand matching algorism is implemented using these functions. As a result, the proposed framework reproduces the original power supply and demand status from the 2011 Great East Japan Earthquake in a relatively consistent manner. Using stochastic components of the statistical functions, the average supply margin is estimated to be 4.1%, but the worst-case scenario is a 5.6% shortfall relative to peak demand. Thus, by applying the framework, the study improves knowledge on potential risk by examining a particular past disaster; the findings are expected to enhance risk perception and supply and demand preparedness after a future large-scale earthquake and tsunami disaster.

## Introduction

In the past decade, large-scale power outages have occurred due to natural hazards, such as Hurricane Sandy in 2012 [1] and the Texas electricity crisis in 2021 [2]. Rudnick [3] describes how natural disasters and their impact on electric power system functioning have become of increasing interest to countries worldwide. Long-term power conservation is sometimes required, particularly in large-scale natural disasters during which power plants and trunk transmission systems are damaged. The Great East Japan Earthquake [4] and the 2018 Hokkaido Eastern Iburi Earthquake [5] are typical examples of such events, especially the 2011 Great East Japan Earthquake, which resulted in power shortages for at least five months. Ministry of Economy, Trade and Industry (METI) [6] focused on the 2011 Great East Japan Earthquake and presented a discussion of long-term power shortage problems for future large-scale earthquake scenarios; our prior preliminary methods were based on this discussion, but a more practical model was needed for further consideration.

An increasing number of studies have examined electric power systems, which typically exhibit the most issues among many critical infrastructures, especially regarding the problems of power outages and restoration during disasters. Specifically, achieving resilience, which minimizes losses incurred by society as a whole, is a key concept in this type of analysis [7]. One approach to tackle this issue is modeling the recovery processes of both power supply and demand, which is the subject of this study, and has become a major issue in recent years. For example, Sun et al. [8] and Didier et al. [9] worked on the power supply shortage risk after a disaster considering the damages of both the supply and demand sides. In these studies, the recovery of power consumption is estimated by assuming the probability distributions of recovery dates for different types of damaged buildings, such as an apartment, hospital, school, and industrial unit. These studies provide a fundamental idea that power shortage risk requires modeling the recovery of society after a disaster as well as the recovery of supply capacity of power systems.

However, these previous studies focused on relatively short-term power shortages. For example, Sun et al. [8] assumed a hypothetical community which is hit by a large earthquake, where power supply meets demand within two weeks after the earthquake. Didier et al. [9] analyzed the power shortages after the 2015 Gorkha earthquake in Nepal. In their simulation model, the power system failure was resolved within nine days. This is natural because most power outages caused by real disasters are relatively short-term (e.g., most cases considered a single day, while a small number of cases considered more than a day but at most two weeks with respect to major blackouts worldwide, during 2006–2012 [10]); long-term supply-demand gaps have not been fully studied, such as those caused by the Great East Japan Earthquake.

The purpose of this study is to provide a framework for analyzing such a long-term supply and demand gap after the disaster, adapting statistical functions on the vulnerability and resilience characteristics of power supply and demand systems, and to apply it to the Great East Japan Earthquake. These functions have been partially developed using past disaster data sets including the Great East Japan Earthquake, but have never been integrated for the analysis of power shortages. For example, the model for power demand recovery is proposed based on the business recovery model after the disaster [11]. The damage and recovery functions of thermal power plants have been determined from a previous study, and the supply capability of the total power system is evaluated by integrating the damage and recovery functions of components in a trunk distribution system. Finally, as a method for balancing power supply and demand, this study adopts a relatively simple algorithm that considers the constraints on generation and transmission capacity to investigate the adequacy of the power system. Through the application, the proposed framework reproduces the original power supply and demand status from the 2011 Great East Japan Earthquake in a relatively consistent manner and estimates the average supply margin to be 4.1%, but with a 5.6% shortfall relative to peak demand in the worst-case scenario.

## Materials and methods

### Supply capacity recovery

Large-scale earthquakes that generate strong ground motions may extensively disable substations and power transmission lines. In general, power supply routes are multiplexed to avoid large-scale power outages if one such facility is damaged. This is called the N-1 rule, which indicates that the failure of one out of N facilities does not affect the functionality of the power system [12]; in many cases, N-2 or greater damage (more than two facilities are damaged) can also be absorbed by the system overall. However, if the damage exceeds a certain acceptable level for a certain period of time, depending on the structure and operational mode of the power supply systems, a large-scale power outage occurs. Therefore, the possibility of damage and the expected time to restore substations, transmission lines, and power plants are necessary to understand supply capacity after an earthquake. In this study, the damage and restoration time of these facilities were estimated using fragility and restoration functions, based on previous research [13, 14].

To estimate the recovery time of thermal power plants, a two-step evaluation approach was used, which considered seismic and tsunami fragility curves (log-normal function) and a recovery function based on the experience data of 29 thermal power plants affected by the Great East Japan Earthquake [13]. In the first step, damage occurrence and its degree for each typical plant system (e.g., boilers) were estimated via fragility curves. Three ranks of damage degree are set as follows: Severe: collapse or major repairs required; Moderate: partial damage, inundated, or partial repair required; and Minor: minor defects (e.g., small deformation). In this study, it was assumed that the occurrence of damage due to ground motion and that due to tsunami were independent; then, the probability of damage to the *n*th facility was an arbitrary rank *k* at a given peak ground acceleration (PGA) and tsunami inundation depth. *P*_*n*,*k*_ was calculated as follows (1):

$$P_{n,k}({PGA}_{i},{ID}_{i})=P_{1,n,k}({PGA}_{i})+P_{2,n,k}({ID}_{i})-P_{1,n,k}({PGA}_{i})∙P_{2,n,k}({ID}_{i})$$ (1)

where *P*_1,*n*,*k*_ is the probability that the damage rank of the *n*th facility due to ground motion is greater than or equal to *k*

$$P_{1,n,k}=\Phi \left(\frac{lnPGA-\mu_{1,n,k}}{\sigma_{1,n}}\right)$$ (2)

where *P*_2,*n*,*k*_ is the probability that the damage rank of the *n*th facility due to a tsunami is greater than or equal to *k*

$$P_{1,n,k}=\Phi \left(\frac{lnID-\mu_{2,n,k}}{\sigma_{2,n}}\right)$$ (3)

where *PGA*_*i*_ is PGA at plant *i* [gal] and *ID*_*i*_ is inundation depth at plant *i* [m]

This is a typical simplified model of facility damage. Similarly, the fragility curves for other facilities were obtained. The recovery functions of thermal power plants and distribution systems were also based on observations from disasters [15, 16]. The detailed formulae and parameter values are summarized in **S1 Appendix**.

### Demand recovery

To evaluate electricity demand after a disaster, it is essential to appropriately model the socioeconomic recovery situation, which is highly correlated with electricity demand. However, model construction to evaluate the socioeconomic recovery process after a disaster is a challenging task; it is necessary to develop a model structure and rules for estimation while utilizing data obtained from existing disaster cases [11].

Nevertheless, methods for analyzing the socioeconomic impact of disasters continue to progress, and in this study, we used a set of models to evaluate the process of post-disaster socioeconomic recovery. In particular, the power demand of the industrial sector changes drastically during a disaster. This is largely due to damage to corporate facilities and shortages of raw materials and lifeline supplies necessary for production. For example, following the Great East Japan Earthquake, industrial activities were suspended over a wide area, and it took a long time for heavily damaged industries to recover.

Conversely, for the household sector, we assumed that a significant drop in electricity demand did not occur, except in areas with power outages or tsunami-affected areas, because electricity service is essential for daily life (e.g., lighting, laundry, and cooking), including after a disaster. Therefore, we reflected only the power outage effect in our power demand model for the household sector. It is likely that power demand increases at evacuation points, but these effects were assumed to be a minor proportion of total consumption. Rather, the power-saving rate during the crisis would be more dominant. Fujimi et al. [17] estimated household power savings in the summer of 2011 as 12.8% for Tokyo Electric Power Company (TEPCO) and 9.4% for Tohoku Electric Power Company (THEPCO). Because power-saving activities for both household and industrial sectors were not modeled in our study, a discrepancy was noted between the observed and estimated power demands in a later analysis of consumption during summer.

**Fig 1:**
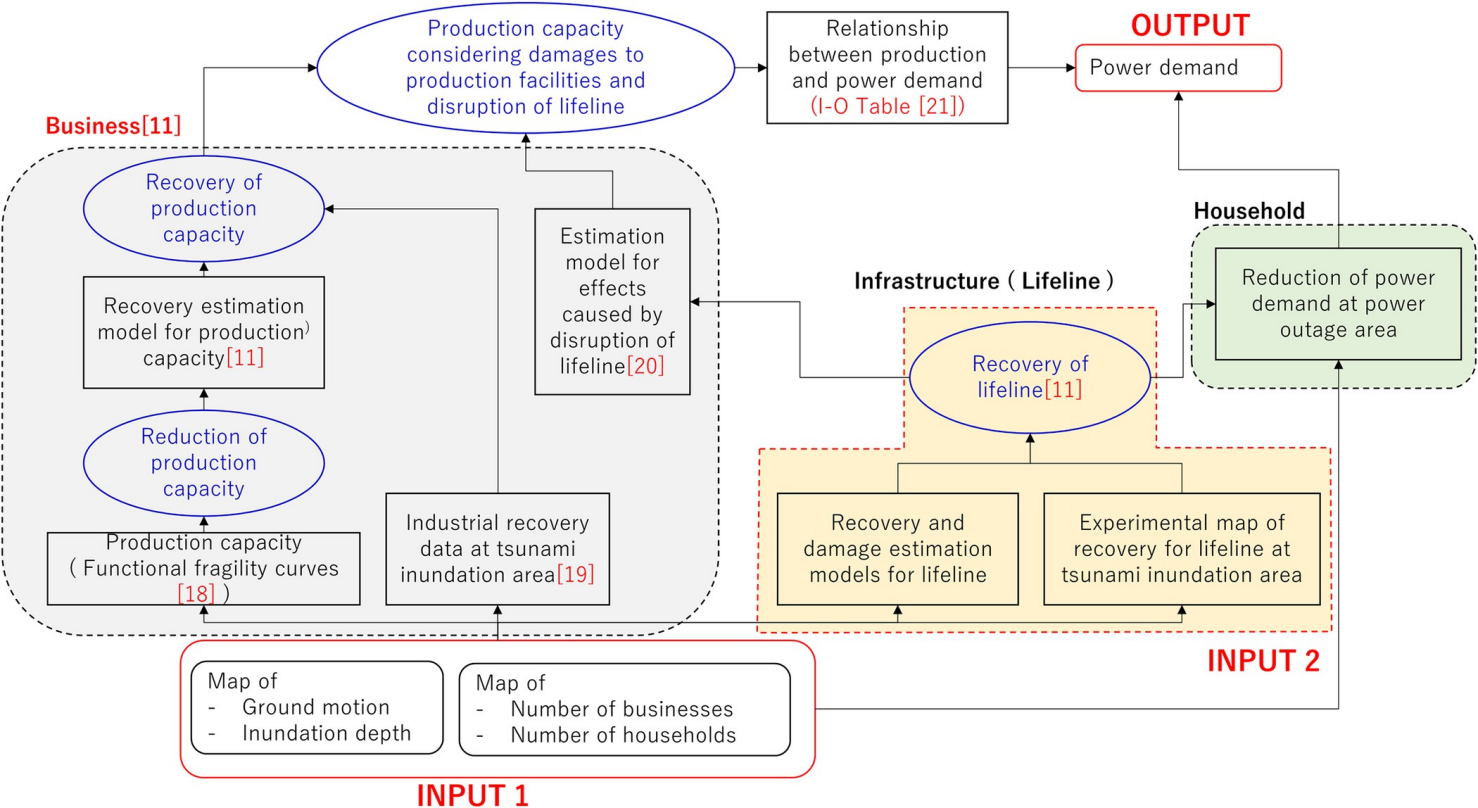
Framework for estimating power demand recovery.

Fig 1 shows the framework for estimating electricity demand under the disaster conditions used in this study. The model assumes electricity demand during disasters in two sectors, business establishments and households, and the main model is a recovery forecast for electricity demand in establishments, which has particularly high demand. First, as inputs, the model uses information on hazard (earthquake motion and inundation by tsunami) and exposure (number of employees at establishments classified into 26 industries and number of households). Data for both hazard and exposure are maintained in 500 ⅿ mesh basis (INPUT 1). Hazard information can also be used to predict lifeline (electricity, water, and gas) restoration using engineering models, but this study uses observed data on the number of days of lifeline restoration in each region during the Great East Japan Earthquake [11] (INPUT 2).

The business aspect (left box in Fig 1) represents the direct impact of the hazard on production capacity and the indirect impact through lifelines disruption. For direct impacts, the functional fragility curve introduced in Nakano et al. [18] is used to estimate the percentage of reduction in production capacity based on the intensity of ground motion at each site (500 m mesh). This production capacity primarily reflects functional damage to facilities and the difficulty in mobilizing employees. For the subsequent recovery process, the authors applied the average production capacity recovery curve [11] obtained from a questionnaire survey after the Great East Japan Earthquake, conducted by the authors for areas that were not damaged by the tsunami, and the same survey results were applied for the tsunami-affected areas [19]. Furthermore, for business, the impact of reduced production capacity due to lifeline outages is taken into account as an indirect impact [20]. The procedure of integrating the above processes is detailed in a previous study [11].

Meanwhile, this study needs to estimate the recovery of electricity demand, not production capacity. In other words, the faster the recovery of electricity-intensive industries, the faster the overall recovery of electricity demand. To reflect these industry-specific demand characteristics, data on power usage per unit production for different industrial sectors based on the 2005 input-output table [21] were used to convert the production level to the power consumption level in the industrial sector. For household electricity demand (right side of Fig 1), only the reduction in electricity demand in blackout areas is considered, as mentioned earlier.

The scope of this demand assessment was limited to areas where companies suffered direct damage to their facilities. We consider this the dominant factor underlying economic suspension in the Tohoku region, but, as we observed, the damage spread to the economies of other regions.

### Supply-demand balancing model

The supply-demand balancing algorithm was constructed from the perspective of adequacy. The capacities of both generators and transmission lines became restrictive conditions. Supply and demand were matched in an auction style, in which the shorter distances discounting transmission losses were given higher priority for determining supply and demand node pairs. This provides a feasible solution to the assignment of power supplies from different generators meeting demand, considering transmission capacity. In traditional network analysis, the algorithm has been used to solve for minimal cost flows [22].

**Fig 2:**
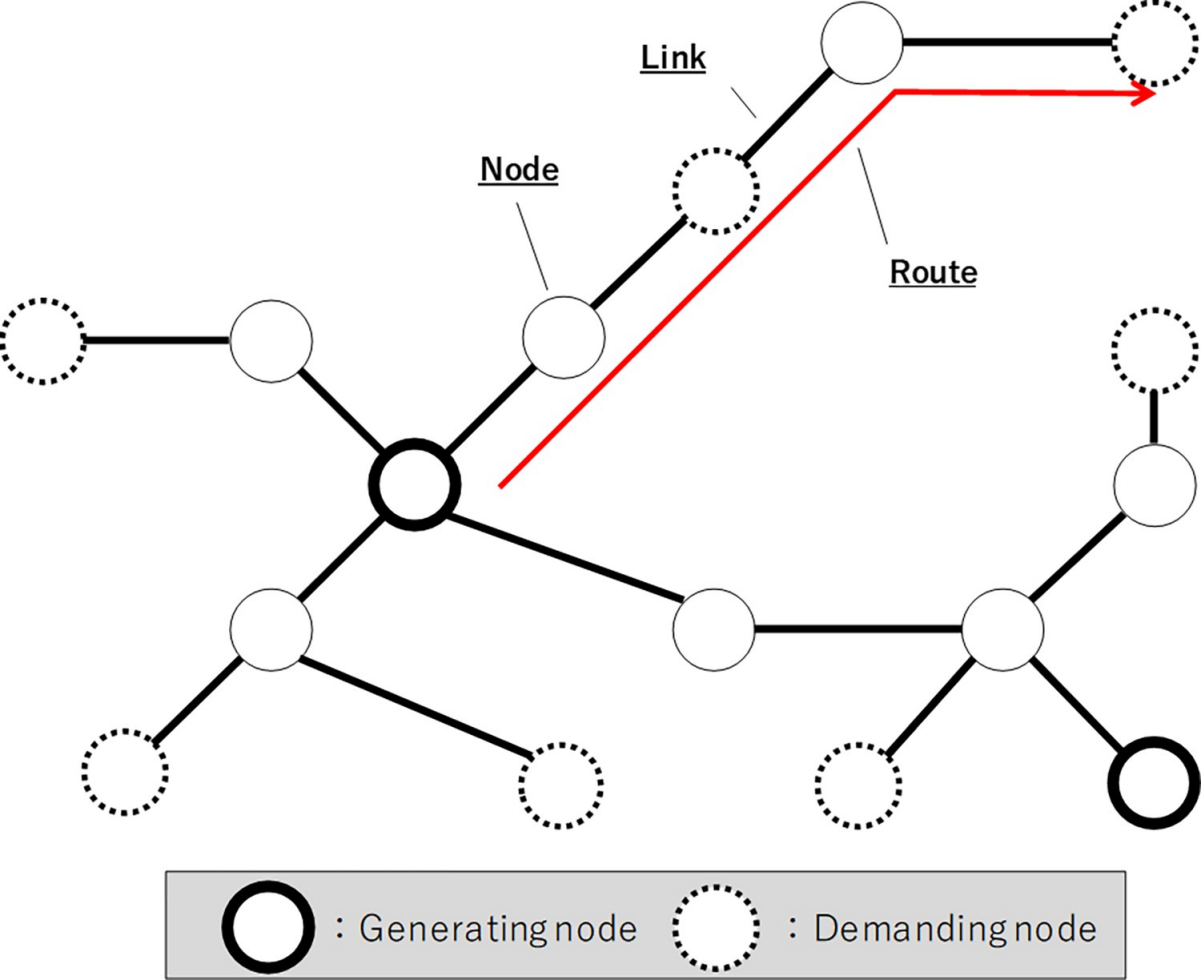
Definition of nodes, links, and routes.

In the current study, the target systems were primary power plants, transmission lines, and major substations (over 154 kV). Fig 2 shows the simplified structure of the power systems assumed in this study, wherein power plants and primary substations, ultra-high voltage substations, and switchyards are represented by nodes, and power transmission lines are expressed by a link connecting node pairs. In particular, we call the node representing the power plant the "power generation node,” and the node representing the primary substation the "demand node." We also call the transfer capacity set for each link the "link capacity,” and the transfer capacity in the route from the power generation node to the demand node the "route capacity." The route capacity is equal to the minimum value of the link capacity of all links on the route. The distance of each link is the sum of the span lengths between the towers included in the link, and we name the sum of the distances of all the links existing on the route the "route distance." The method involves simply matching the demand node with the power generating node using the shortest route under the constraint of link capacity. This is a typical network flow assignment problem [23] and more details are provided in **S2 Appendix**.

### Data set

**Fig 3:**
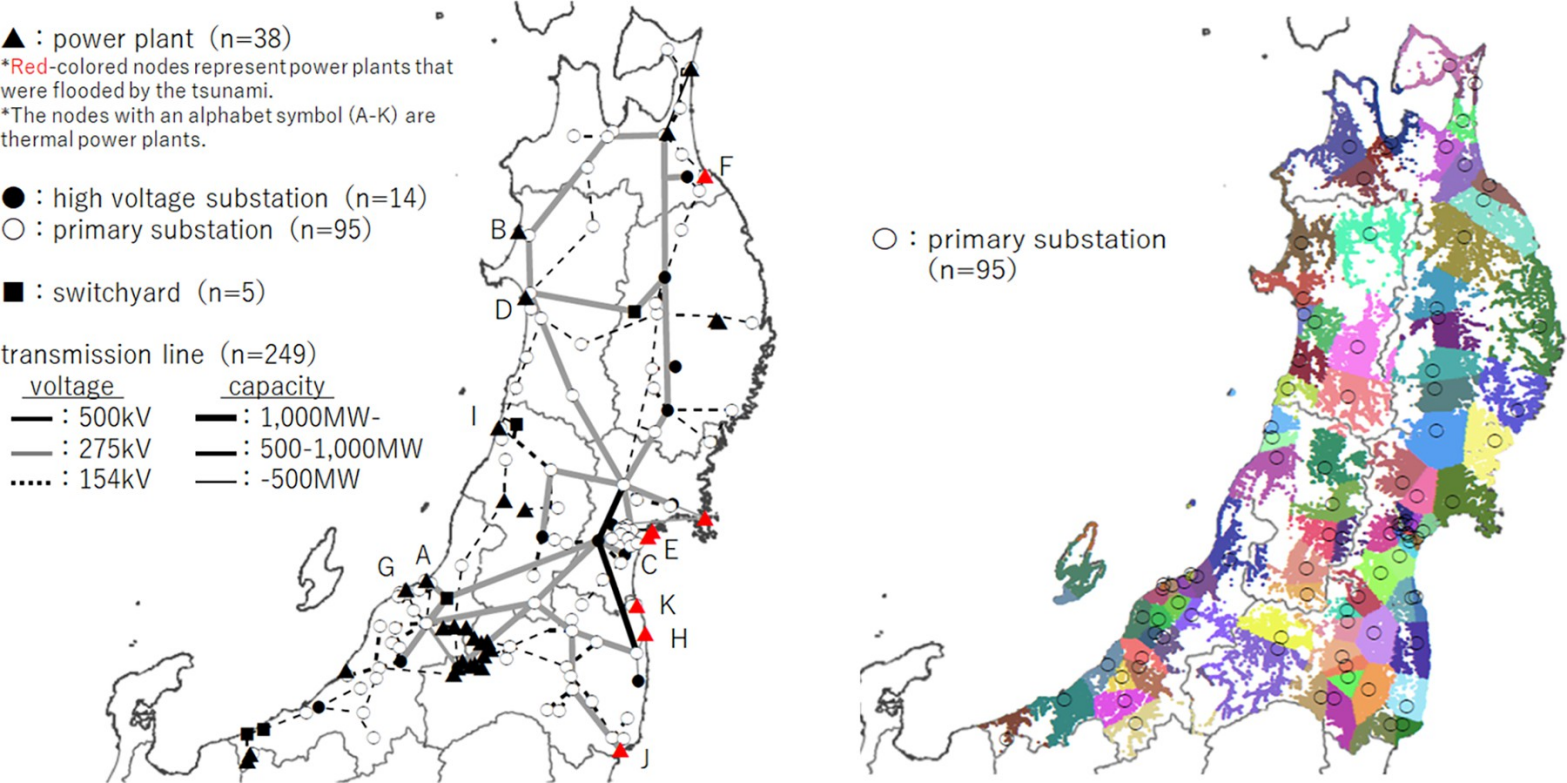
Power system and supply areas in Tohoku region, Japan. **a**: Power system diagram used in the simulation. **b**: Distribution of primary substations and supply territory (color is intended to visualize the supply area boundary; each color has no particular meaning. The map of Tohoku and other regions with prefectural boundaries are obtained from [25]).

Fig 3 presents the power system diagram and supply area of each substation used in the simulation. The total number of nodes was 195, of which 38 were power plant nodes, 109 were substation nodes, five were switchyard nodes, and 43 were transmission line branch points. Among the power plant nodes, there were 11 thermal power plant nodes, 22 hydroelectric power plant nodes, three nuclear power plant nodes, and one geothermal power plant node. In addition, transfer points receiving power from other electric power companies were treated as power plants in the simulation. We defined substation nodes with a secondary side greater than 66 kV as “high voltage substations” and those less than 66 kV as “primary substations.” Based on this classification, 109 substations were divided into 14 high-voltage substations and 95 primary substations; the latter were set as demand nodes. In Fig 3, supply area boundaries were estimated by allocating each business location [24] to the closest substation.

The total number of links was 249, and there were transmission towers in each link. The total number of transmission towers was 15,654, and the maximum number of transmission towers in one link was 370. More details of the system setting and hazard information are provided in **S3 Appendix**.

## Results and discussion

### Estimated demand at each node

**Fig 4:**
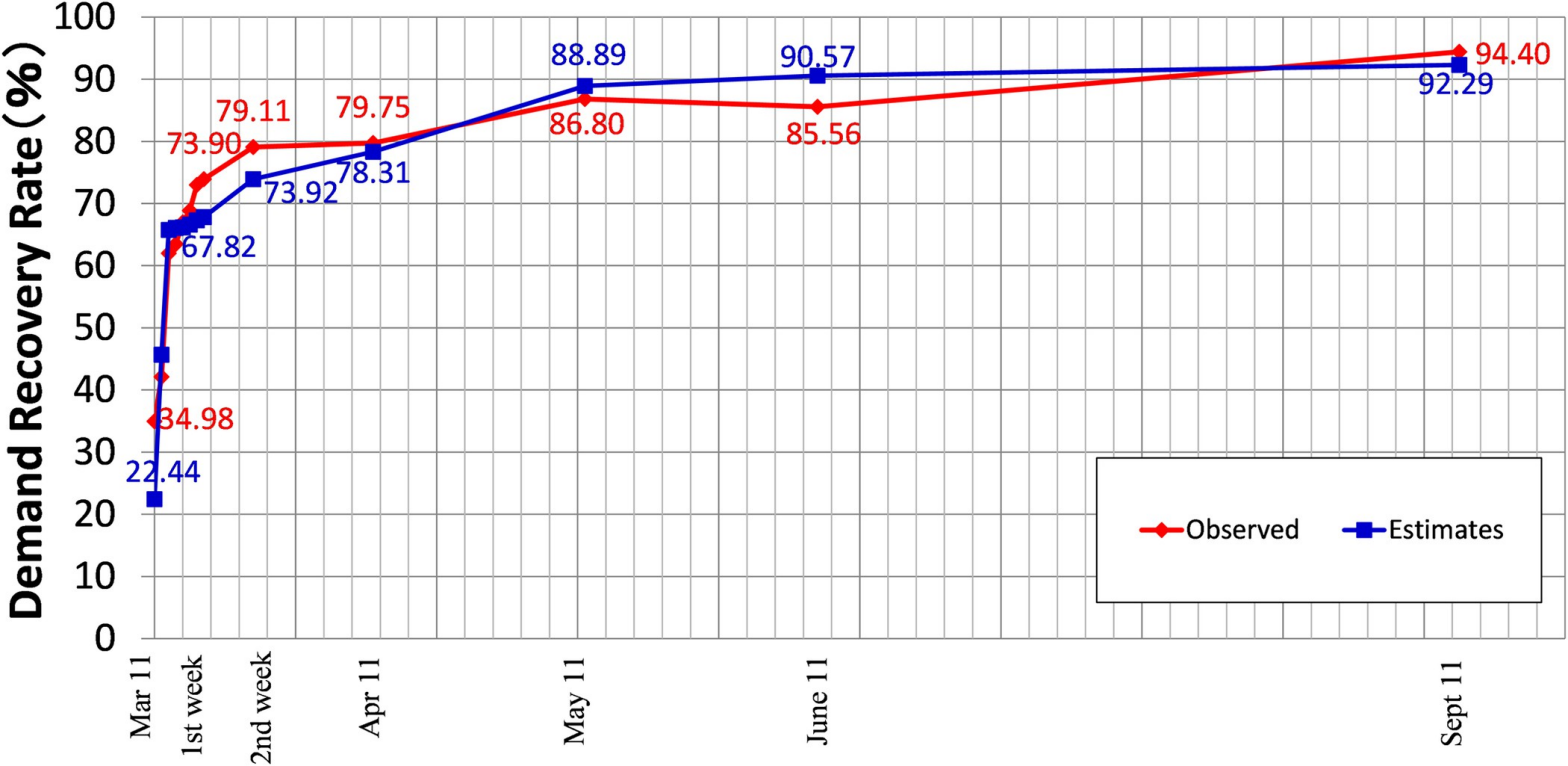
Validation of the power demand model. The observed data in 2011 were standardized by the electricity demand in 2010. The figure illustrates the power demand in the overall area. The grid interval is four days. The power demand at each substation was also obtained by aggregating the 500 m grid scale to the appropriate substation territory shown in Fig 3.

A power demand simulation after the Great East Japan Earthquake was carried out. Fig 4 shows the results derived from estimating the electricity demand recovery process. An advantage of the approach used here is its ability to estimate the power demand at a relatively detailed spatial scale based on hazard and lifeline disruption information.

In Fig 4, the observed data in 2011 were standardized by the electricity demand on the “close date” in 2010 [26]. We used the close date because of adjustment for holidays. January 1, 2010, was a Friday, while January 1, 2011, was a Saturday. Therefore, we adopted a time series in 2010, starting from January 2. Because we were interested in the peak demand, the average power demand during 15:00–16:00 was employed to compare two time series. In this time slot, solar power may contribute to the supply capacity, but the installed amount of solar power panels was small at the time of the disaster.

Fig 4 demonstrates that the estimates follow the observations relatively well. At maximum, an underestimation slightly over 5% was generated two weeks after the disaster, probably because of the longer estimates of lifeline disruption periods. A 5% overestimation was observed on June 11, three months after the disaster, when many people and industries commenced maximum efforts to save power during the summer season. For the later supply-demand balance case studies, we used this model for the power demand at a 500 m grid scale, but adjusted to fit the observations in the total supply area multiplied by a constant scale factor.

### Supply-demand balance and potential scenario analysis

The stochastic components of the supply-demand balancing model in this study were damage occurrence and the number of days of recovery for thermal power plants, which accounted for most of the power supply. Based on the probability distributions of these stochastic components, 2,500 Monte Carlo simulations were performed.

**Fig 5:**
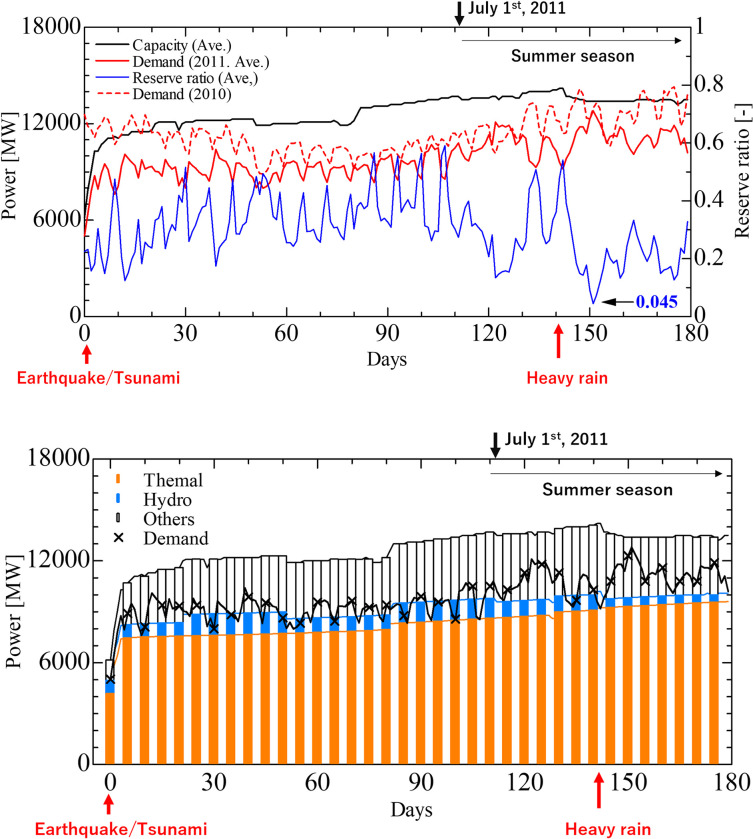
Average of 2,500 supply-demand simulation results. **a**: Time series of supply capacity, demand, and reserve ratio. **b**: Time series of power supply by generation type.

First, we examined the trend of the average of the 2,500 simulation results. Fig 5A shows the post-disaster time series of the supply capacity, demand, and reserve ratio of the entire target power system. Here, the reserve ratio was defined as the daily minimum ratio of the reserve power (supply capacity minus power demand) to the power demand [15]. The actual demand in the previous year (2010) is also indicated by a dotted line. Fig 5B illustrates the time series of supply and demand for each of the three types of power generation: thermal, hydro, and other. These results are relatively consistent with the observed supply capacities [15].

The average power supply dropped from 14,000 to approximately 6,000 MW immediately after the earthquake, but recovered to approximately 10,000 MW in approximately three days. This was due to restoration of the thermal power plant units, which had automatically shut down for safety immediately after the disaster. Since then, thermal power plants recovered slowly, while hydropower plant supply increased and decreased repeatedly. In addition, heavy rains on day 141 intensively damaged the hydraulic power plants and reduced their power supply.

A comparison of the average supply capacity and demand revealed that the supply capacity always exceeded demand, and the reserve ratio was greater than zero. However, Fig 5B shows that the demand was larger than the sum of the capacities of the thermal and hydroelectric power plants, which is regarded as the primary supply capacity. Shortages are covered by geothermal power plants, private power generation, and regional transfers. The minimum value of the reserve ratio was 0.045, which is assumed to be caused primarily by an increase in demand in summer under the supply capacity constraints, due to damage to thermal power plants by a tsunami in March and damage to hydroelectric power plants by heavy rains in July, 2011 [15]. In the verification of the supply-demand gap by METI [16], the minimum value of the reserve ratio was 0.039 during August, even if the regional transfer was 1,700 MW from other companies. This is worse than the average case scenario.

Compared with the previous year’s demand, the demand in the simulation was generally smaller, indicating significant demand-side damage and power-saving effects. However, during the peak demand period in the summer around the 150th day, the supply and demand balance was tight, indicating that the situation was extremely severe.

**Fig 6:**
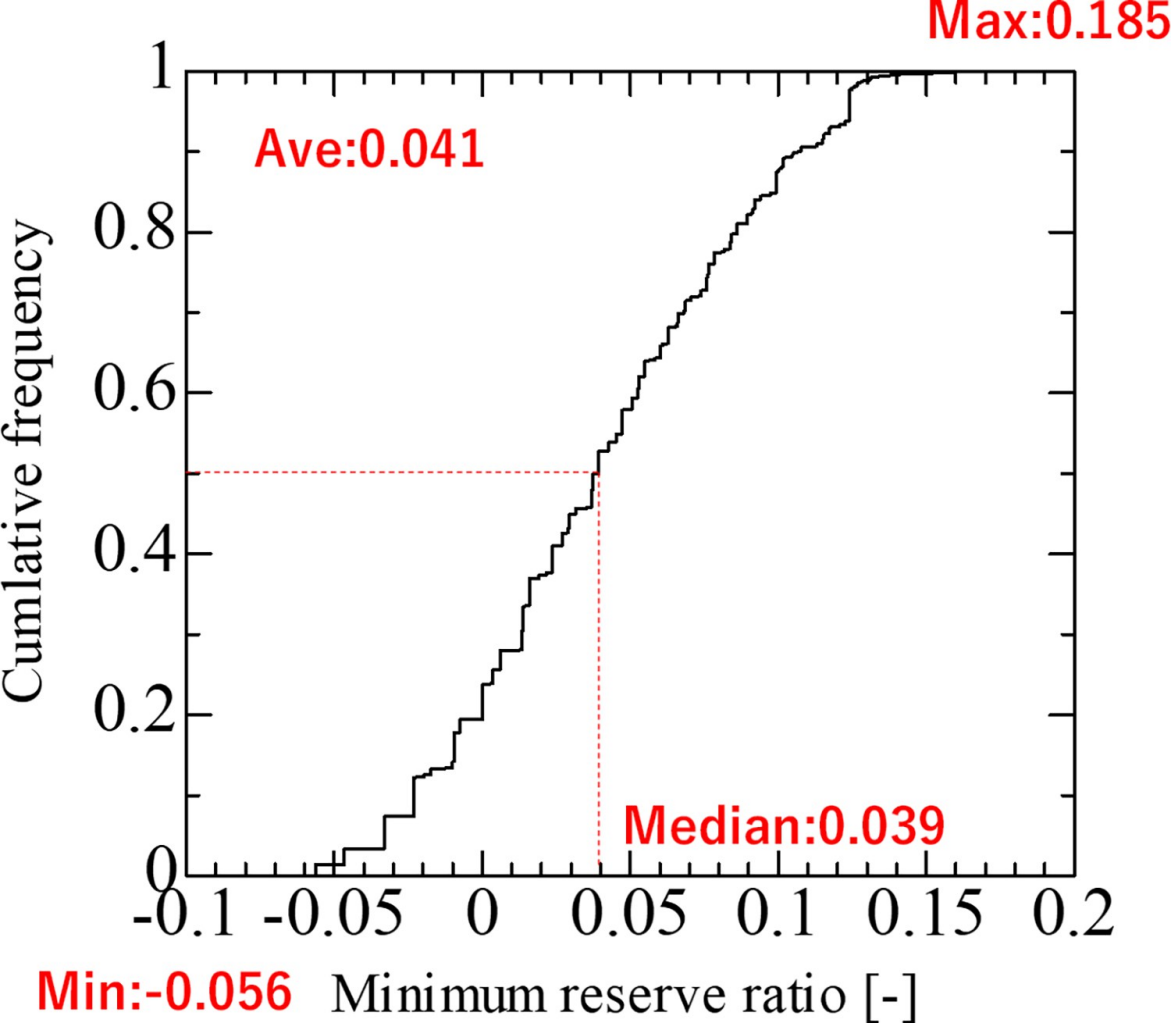
Cumulative distribution of the minimum reserve ratio.

Next, we investigated the typical worst and best cases. Based on these results, we defined the worst case as that with the smallest minimum reserve ratio and the best case as that with the largest minimum reserve ratio, and analyzed the supply-demand balance for each. Fig 6 shows the cumulative frequency distribution of the minimum reserve ratio. The minimum reserve ratio was -0.056, the maximum was 0.185, the mean was 0.041, and the median was 0.039.

**Fig 7:**
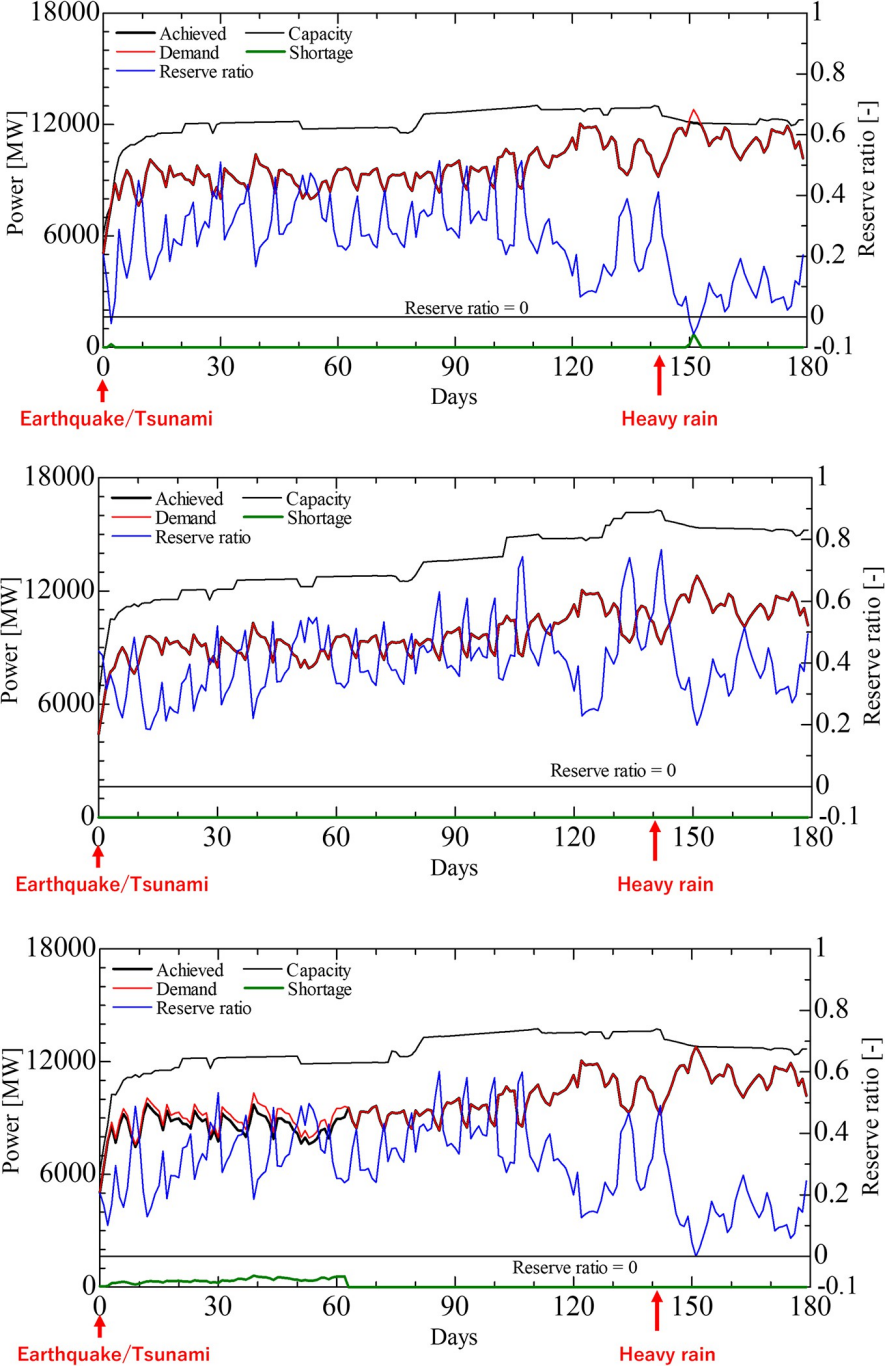
Comparison of the cases with the smallest and largest minimum reserve ratio. **a**: Case with the smallest minimum margin. **b**: Case with the largest minimum margin. **c**: Case with the trunk transformation system damaged.

Fig 7A and 7B show the time series of supply, capacity, demand, power shortage, and reserve ratio for the cases with the smallest and largest minimum margins, respectively. In the smallest margin case (Fig 7A), the restoration of thermal power plants was delayed, resulting in power shortages in the summer, and the reserve ratio became negative. In addition, the reserve ratio was below 0.05 in approximately half of the summer months, indicating tight supply versus demand. If the timing of the disaster coincided with these peak periods, the supply-demand gap could have been very severe. In the largest margin case (Fig 7B), there was no power shortage throughout the period, and the reserve ratio was always above 0.2, indicating that the system had sufficient supply capacity.

Finally, we investigated the case in which the cumulative power shortage was the largest. Here, cumulative power shortage is defined as the sum of daily power shortages from disaster occurrence to 180 days later. The time series of supply capacity, power demand, power shortage, and reserve ratio for this case are shown in Fig 7C, which illustrates that the reserve ratio in summer was low, but not negative. However, during the first 63 days after the earthquake and tsunami, a power shortage occurred continuously. The daily simulation results indicated that this is because the substations that serve as hubs in areas where demand is concentrated were damaged, which is one of the scenarios in which power shortages occur, because of damage to transmission and substation facilities, even if supply is sufficient to meet demand. In addition to this case, we also obtained a case in which a substation that acted as a hub for the entire grid was damaged, resulting in power shortages of approximately 30% of demand.

## Conclusions

This research proposed an integrated framework for estimating long-term power shortages after a large-scale earthquake and tsunami disaster, especially focusing on the Great East Japan Earthquake. This type of integrated modeling is vital for improving the resilience of complex critical infrastructures, namely the power system, for identifying system bottlenecks and determining the effects of collective efforts on both supply and demand.

In this study, various large-scale damage data sets after past earthquake and tsunami disasters were adapted for constructing statistical functions on the vulnerability and resilience characteristics of power supply and demand systems. Specifically, the damage and recovery functions of thermal power plants, trunk distribution lines, and businesses were developed as primary components of the overall framework. These functions were then integrated to simulate power shortages by simple supply and demand balancing algorithms. The result, which focuses on the average of 2,500 Monte Carlo simulations, reproduced the original power supply and demand status from the 2011 Great East Japan Earthquake in a relatively consistent manner.

The Monte Carlo simulations also revealed various potential risks, including a case in which power transmission and substation facilities became bottlenecks, and a case that illustrated that supply-demand balance can tighten during summer: a day with a -5.6% reserve ratio during summer as a worst-case scenario even if the mean value was 4.1%. Thus, the developed framework and statistical functions have good potential to simulate benchmark damage and recovery estimations of power systems and reveal potential power shortage risks, especially in the case that the prolonged impacts continue after the earthquake and tsunami.

Regarding policy implications, the model and its results can be used as a communication tool between power suppliers and users. Simulation results can be shared easily with the general public and help determine power dispatching strategies, power usage restrictions, or additional efforts needed to provide an acceptable level of risk. However, as power systems are extensive, future studies have many opportunities to enhance the approach. For example, both post-disaster power supply and demand models are relatively primitive and have limited application to a specific disaster. More data sets are necessary to upgrade the model and validate the effectiveness of the current framework for other disasters. The power system itself has been changing due to the installation of new generators, especially renewable sources, and upgrading the capacity of transmission lines. Based on these data limitations and dynamic system changes, the following are current issues that should be considered.

A supply-side model should include recent trends in renewable energy installation. Eighteen percent of Japan’s power supply is obtained from renewable energy (hydro, biomass, geothermal, solar, and wind power) [27]. Renewable energy has become a major source of power supply and is expected to become increasingly important in the future. Risk and resiliency assessments of renewal sources are therefore highly important. For example, Van der Wiel [28] investigated if weather conditions can jeopardize energy supply security.

In this way, a resilient post-disaster power supply system was discussed from both the supply and demand perspectives. Modeling techniques are essential, but database development, especially recording past large disaster events, is also vital for further explorations. We also hope that the framework of systems analysis could be applied to international cases and other disasters, such as large-scale hurricanes and floods, by adapting damage and recovery functions to these disasters.

## Supporting information

S1 Appendix The detailed formulae and parameter values of damage and recovery of thermal power plants. (PDF)

S2 Appendix Details of the supply-demand balancing model. (PDF)

S3 Appendix Details of the system setting and hazard information. (PDF)

S1 Dataset (ZIP)
