# Supplementary material for: A framework to estimate a long-term power shortage risk following large-scale earthquake and tsunami disasters
Source: PLoS One. 2023 Mar 27;18(3):e0283686. doi: 10.1371/journal.pone.0283686 (PMC10042361; doi:10.1371/journal.pone.0283686)
Supplement: S1 Appendix — (PDF) [file pone.0283686.s001.pdf]

Appendix A (The detailed formulas and parameter values of damage and recovery of thermal power plants)

Table A.1 lists the values of the parameters for the fragility functions for each facility constituting thermal power plants. As is typical in Monte Carlo simulations, the degree of damage to each facility was determined using random numbers, assuming that the damage to each facility was independent of other facility conditions. After estimating the damage to each facility, the recovery period T was determined by the probability distribution given in Eq.(A.1). Parameter values were estimated from the records of the 2011 Great East Japan Earthquake. More details are provided in Yuyama and Kajitani [A1].

$$G(t|X) = \Pr(T \leq t|X) = \Phi\left(\frac{l\,nt-\beta X}{0.54}\right) \quad \text{(A.1)}$$

where β is the parameter of recovery function, $\beta = [1.12 \quad 1.22 \quad 1.41 \quad 1.30 \quad 1.74]$; X is the damage status of facilities that comprised plant systems,

$X^T = \{x_{n,k}\} = [x_{1,C} \quad x_{2,C} \quad x_{3,B} \quad x_{4,C} \quad x_{5,A}]$

where $x_{n,k}$ is binary variable that denotes whether the damage rank of the nth facility was greater than or equal to k (1) or not (0).

Table A.1. Parameters of the fragility function (thermal power plant). a: seismic fragility parameter (unit: PGA[gal]), b: tsunami fragility parameter (unit: inundation depth[m]).

a

n	1: water inlet/outlet facilities	2: power receiving facilities	3: boiler facilities	4: flu gas treatment facilities	5: service buildings
$\mu_{1,n,A}$	—	—	—	—	—
$\mu_{1,n,B}$	5.89	5.94	5.39	6.26	—
$\mu_{1,n,C}$	5.50	5.59	4.87	6.02	—
$\sigma_{1,n}$	0.65	0.34	0.58	0.20	—

b

n	1	2	3	4	5
$\mu_{2,n,A}$	1.08	2.07	2.49	1.53	2.39
$\mu_{2,n,B}$	-0.03	-0.34	0.25	1.31	-0.07
$\mu_{2,n,C}$	—	—	—	1.09	—
$\sigma_{2,n}$	0.80	1.29	0.85	0.34	0.75

"—" indicates that parameter estimation did not occur due to lack of observations.

Damage to a transformer, circuit breaker, and disconnector was estimated, and the damage

to at least one of these facilities was assumed to result in the failure of the substation. This is due to a limitation of the current database. If a substation had back-up facilities, the substation may have not lost total functionality.

Functional damage to a transmission line was determined by the damage to the transmission towers constituting the transmission line. The transmission line itself was unlikely to be damaged by the earthquake ground motion. The transmission tower was assumed to be composed of three facilities: tower body, electric cables, and insulators. The occurrence of damage was estimated for each facility. Similar to the substations, any damage to the facility led to damage to the transmission tower.

In Takabatake et al. [A2], the fragility functions for each component of substation and transmission tower were estimated on the basis of the actual damage data from the 2011 Great East Japan Earthquake and the 2016 Kumamoto Earthquake (5026 for a transformer, 7284 for a circuit breaker, 17167 for a disconnector, 56410 for a tower body, 62909 for an electric cable, and 62909 for an insulator) using the lognormal distribution in Eqs. (A.2) and (A.3).

$$F_X(x) = \int_0^x f_X(x)dx \tag{A.2}$$

$$f_X(x) = \frac{1}{\sqrt{2\pi}(\zeta x)} \exp\left[-\frac{1}{2}\left(\frac{\ln x - \lambda}{\zeta}\right)^2\right] \tag{A.3}$$

where x is the instrumental seismic intensity, and λ and ζ are the parameters of the lognormal distribution. The values of the parameters for each facility are shown in Table A.2. Fig. A.1 shows the fragility function for the transformer, one of the component facilities of a substation.

Table A.2 Parameters of the fragility function. a: substation, b: transmission.

a

	Transformer	Breaker	Disconnector
λ	8.41	10.65	8.38
ζ	1.32	1.84	1.23

b

	Tower	Cable	Insulator
λ	10.44	9.50	11.62
ζ	1.50	1.04	1.98

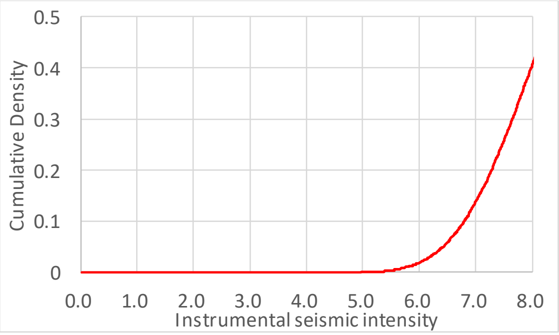

Fig. A.1. Fragility function for transformer.

The restoration times of substations and transmission lines were evaluated using the methods reported by Takabatake et al.,[11] who estimated the restoration functions on the basis of observations from the Great East Japan Earthquake and proposed the following functional form:

$$P_r = exp(-ae^{-bt}) \tag{A.4}$$

where, t is the restoration time [h], and P_r is the probability that the restoration time is t or less. The values of a and b were 3.4 and 0.079, respectively, for the substation, and 3.5 and 0.068, respectively, for the transmission line.

References

[A1] Yuyama, A. & Kajitani, Y. Estimation of damage and recovery function of thermal power plants based on the data of the 2011 Tohoku Earthquake and Tsunami. *J. Jpn. Soc. Civil Eng. A1* **70**, 664-677 (2014).

[A2] Takabatake, D, Kajitani, Y., Yuyama, A. & Ishikawa, T. Study on simulating power supply and demand during large-scale disaster. *Proceedings of the Ninth Japan Conference on Structural Safety and Reliability (JCOSSAR)*, 57-64 (2019).
