# Supplementary material for: A framework to estimate a long-term power shortage risk following large-scale earthquake and tsunami disasters
Source: PLoS One. 2023 Mar 27;18(3):e0283686. doi: 10.1371/journal.pone.0283686 (PMC10042361; doi:10.1371/journal.pone.0283686)
Supplement: S2 Appendix — (PDF) [file pone.0283686.s002.pdf]

Appendix B (Details of the supply–demand balancing model)

Fig. B.1 shows a schematic diagram of the supply and demand simulation rule. In the simulation, the supply capacity from each power generation node, link capacity, and the power demand of each demand node were predetermined on the basis of the damage to the components of the target power system. From those conditions, power system adequacy was confirmed by investigating power generation adequacy and transmission adequacy. The power shortage was calculated as the sum of power generation shortage and route capacity shortage. Regarding supply to the demand nodes, various rules can be considered, such as setting the priority in descending order of importance of the demand nodes (e.g., center of a city) and the order of decreasing transmission loss to the demand nodes. Here, the importance of the demand node was not considered, but the route distance was calculated considering transmission loss. A demand node was paired with a power generation node with the shortest route distance to the demand node. The shortest distance among all pairs with priority that supply and/or demand was partially or fully determined. Priority of power generation (i.e., which power generation node was selected to meet demand) was empirically determined in our study according to the size, types of generators (e.g., combined cycle), and fuels.

The power flow simulated by this model did not necessarily fit the observed power flows, considering that our aim was to detect bottlenecks associated with supply shortage risks and the amount and duration of shortages over the entire power system. The method is simply matching the demand node with the power generating node using the shortest route under the constraint of link capacity. Many algorithms may be used to find a feasible solution; the following algorithm is relatively simple to implement.

(After simulating power supply capacity and demand recovery at each time period,)

[Procedure 1] Select the generator PGtarget that belongs to the power generation node PPtarget whose generation priority is high.

[Procedure 2] For all demand nodes, calculate the shortest route LPPtarget->Di from the PPtarget and its route distance dPPtarget->Di, excluding links damaged or over capacity.

[Procedure 3] Select the node Dtarget with the shortest route distance dPPtarget->Dtarget among all demand nodes where the power supply has not yet met the demand.

[Procedure 4] The power supply capacity CP(PGtarget) of PGtarget, the remaining power demand RD(Dtarget) of Dtarget, and the route capacity CL(LPPtarget->Dtarget) on the supply route LPPtarget->Dtarget are updated according to the following three conditions.

Case A: CP(PGtarget) ≥ RD(Dtarget) and CL(LPPtarget->Dtarget) ≥ RD(Dtarget)

Dtarget meets supply and RD(Dtarget) becomes zero. In addition, the amount of power supplied to Dtarget, that is, RD(Dtarget), is subtracted from CP(PGtarget)

and the link capacity of LPPtarget->Dtarget.

Case B: [CP(PGtarget) ≥ RD(Dtarget) and CL(LPPtarget->Dtarget) < RD(Dtarget)] or [CP(PGtarget) < RD(Dtarget) and CL(LPPtarget->Dtarget) < RD(Dtarget)].

Because CL(LPPtarget->Dtarget) is insufficient, it is necessary to reselect the route. At this time, the power equivalent to CL(LPPtarget->Dtarget) is supplied to Dtarget so that CL(LPPtarget->Dtarget) is subtracted from CP(PGtarget), RD(Dtarget), and link capacity LPPtarget->Dtarget. The process returns to [Procedure 2].

Case C: CP(PGtarget) < RD(Dtarget) and CL(LPPtarget->Dtarget) ≥ RD(Dtarget)

PGtarget achieves supply to Dtarget and CP(PGtarget) becomes zero. In addition, the amount of power supplied to Dtarget, that is, CP(PGtarget), is subtracted from RD(Dtarget) and the link capacity LPPtarget->Dtarget, and returns to [Procedure (1)].

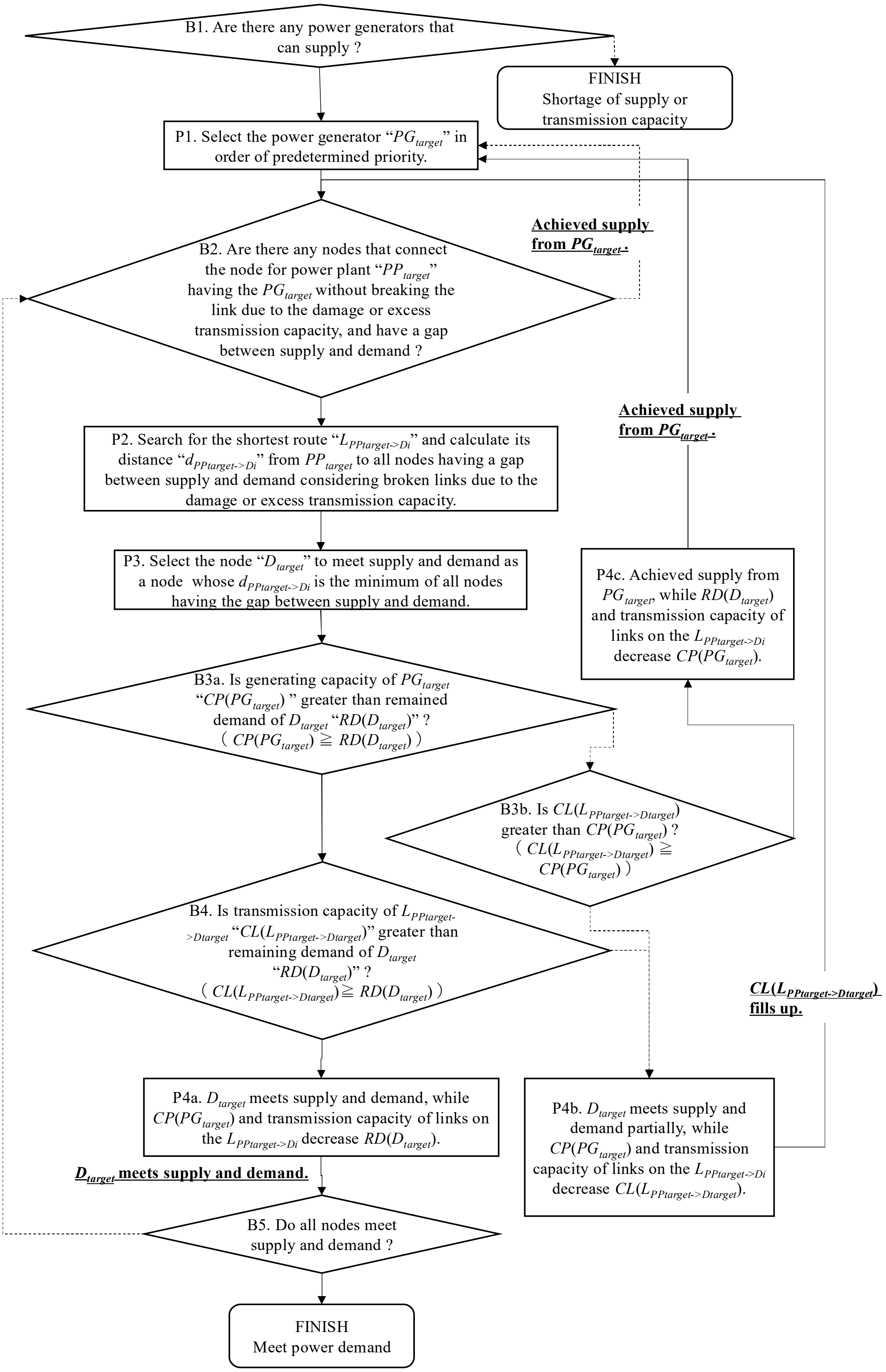

Fig. B.1. Algorithm for balancing supply and demand.
