# Supplementary material for: A framework to estimate a long-term power shortage risk following large-scale earthquake and tsunami disasters
Source: PLoS One. 2023 Mar 27;18(3):e0283686. doi: 10.1371/journal.pone.0283686 (PMC10042361; doi:10.1371/journal.pone.0283686)
Supplement: S3 Appendix — (PDF) [file pone.0283686.s003.pdf]

Appendix C (Details of system setting and hazard information)

In addition to the supply from thermal power plants, hydroelectric power plants, nuclear power plants, geothermal power plants, and conversion plants that were set as generation nodes, the simulation considered the amount of power generation, other than that of power companies. Table C.1 summarizes the sources of supply considered in the simulation and the methods for estimating supply capacities. Probabilistic methods (fragility and recovery models) were applied to estimate supply capacities only for thermal power plants, which had the largest share of supply capacity. The probabilistic components affect the distribution of supply margins, which is a focal point of case studies. As a compromise, the amount of on-site power generation, which was produced by the consumer's own generator, was expressed by uniformly decreasing the power demand of each demand node, as its generation area could not be specified. The observed supply capacity was interpolated from open data provided by the Tohoku Electric Power Company [C1]. The nuclear power plants were stopped during the simulation period. For regional transfer, 600 MW transferred from Hokkaido (north region) was further transferred to Kanto (south region), passing the power network in the Tohoku region before June 2011. Subsequently, 300 MW was transferred from Tokyo I by July 2011 and 600 MW was expected in August 2011. Larger emergency transfer from the Kanto region was frequently observed[1], which is not reflected in the simulation because the small reserve ratios were estimated on different days from the days observed in our simulations, and on these days transfer might not have been available.

The daily amount of self-generation (privately owned generators) was linearly interpolated based on observations[1], because the observed data were not available every day. The supply capacity of hydroelectric power plants was reduced from their maximum capacity based on the monthly average rate of discharge in a river[C2-C9] and damage and recovery status [C1] after heavy rain in July.

Next, for the generation nodes, the power generation priority was set according to the type of power plant, primarily depending on fuel type, which is related to power generation cost. First priority was assigned to power transfers from other power companies, which were treated as a pre-determined exogenous condition. The second priority was for coal-fired power plants, the third for flow-in-type hydropower plants, the fourth for LNG-fired power plants, the fifth for geothermal power plants, the sixth for reservoir-type hydropower plants, the seventh for natural gas-fired power plants, and the eighth for oil-fired power plants. This is an intuitive ordering; these priorities could be updated using an optimal economic dispatching model to evaluate more realistic power flows, but the update was not necessary for this study because our purpose was primarily to investigate power system adequacy, namely whether supply from any power sources meets demand.

Damage caused by the earthquake and tsunami was considered only for thermal power plants. The amount of power supplied by thermal power plants was based on the rated output, but

was set to zero for damaged plants. Some units in thermal power plants are equipped with automatic shutdown devices that can bring the plant to an emergency stop when strong ground motion is detected[10]. Our case study reflected a temporary stop and recovery duration for units that experienced automatic shutdown during the Tohoku earthquake.

Table C.2 shows the rated outputs and fuel used in thermal power plants. The total rated capacity of all power plants excluding nuclear power plants was 14473.63 [MW], of which 12327 [MW] was provided by thermal power plants, 1495.8 [MW] by hydroelectric power plants, and 65 [MW] by geothermal power plants. MW]; other power sources provided 585.83 [MW.

Hydroelectric and geothermal power plants were not severely damaged by the Great East Japan Earthquake. Essentially, the supply capacity of hydropower plants was set according to the actual generation rate. This reflects the severe damage to hydropower plants due to heavy rain during the summer of 2011. Because of heavy rain, the supply capacity was largely lost on July 29, 2011 (the 141st day of the simulation). Capacity loss was not observed in the geothermal power plants.

The nuclear power plant (Onagawa power plant) was not severely damaged by the earthquake and tsunami, but stopped for emergency response, inspection, and recovery. Therefore, the amount of power generated by the nuclear power plant was always set to zero.

Table C.1. Power supply sources and methods to evaluate supply capacity.

Power sources	Methods to evaluate supply capacity
Thermal	Evaluating damage and recovery time from earthquake and tsunami with probability functions
Hydro	Supply capacity is set according to the observations. (No damages occurred during the Tohoku earthquake, but impacts of the heavy rain in July 2011 are reflected）.
Nuclear	Supply capacity is set according to the observations (set as zero).
Geothermal	Supply capacity is set to be the rated output.
Regional transfer	Amount of regional transfer is set according to the observations.
Self-generation	Amount of power supply is set according to the observations, and extract from power demand of each demand note.

Table C.2. List of thermal power plants.

Power Plant	Generator	Rated output [MW]	Generation method	Fuel used	Power Plant	Generator	Rated output [MW]	Generation method	Fuel used
A	1	600	Steam	LNG	E	1	468	C.C.	N.G.
	2	600	Steam	LNG	F	1	250	Steam	Oil
	3	614	C.C.	LNG	G	1	250	Steam	LNG
	4	614	C.C.	LNG		2	54.5	C.C.	N.G.
	5	826	C.C.	LNG		3	54.5	C.C.	N.G.
	6	885	C.C.	LNG		4	34	G.T.	N.G.
	7	350	Steam	LNG	H	1	1000	Steam	Coal
	8	350	Steam	LNG		2	1000	Steam	Coal
B	1	600	Steam	Coal	I	1	350	Steam	Coal
	2	600	Steam	Coal		2	350	Steam	Coal
	3	600	Steam	Coal	J	1	250	Steam	Coal
C	1	350	Steam	Oil		2	600	Steam	Coal
	2	600	Steam	LNG		3	600	Steam	Coal
D	1	350	Steam	Oil		4	250	C.C.	Coal
	2	350	Steam	Oil	K	1	1000	Steam	Coal
	3	600	Steam	Oil		2	1000	Steam	Coal
	4	333	G.T.	Oil					

C.C.: Combined Cycle, G.T.: Gas Turbine, N.G.: Natural Gas

Table C.3 shows the PGA and tsunami inundation depth at the thermal power plant location during the Great East Japan Earthquake. Fig. C.1 and Fig. C.2 show the frequency distribution of

substations and transmission towers for each instrumental seismic intensity. In the supply and demand simulation, the restoration time was estimated via Eqs. (A.1) to (A.4) in **S1 Appdendix**, using the instrumental seismic intensity at the location of each facility and random numbers from 0 to 1. If a transmission route is not damaged, publicly available information on transmission line capacity [C10] is used.

Table C.3. Observed PGA and tsunami inundation depth at thermal power plant sites.

Power plant	PGA [gal]	Inundation depth [m]	Power plant	PGA [gal]	Inundation depth [m]
A	30	—	G	34	—
B	47	—	H	735	13.0
C	512	3.0	I	55	—
D	52	—	J	471	1.8
E	550	4.7	K	585	3.0
F	143	0.5			

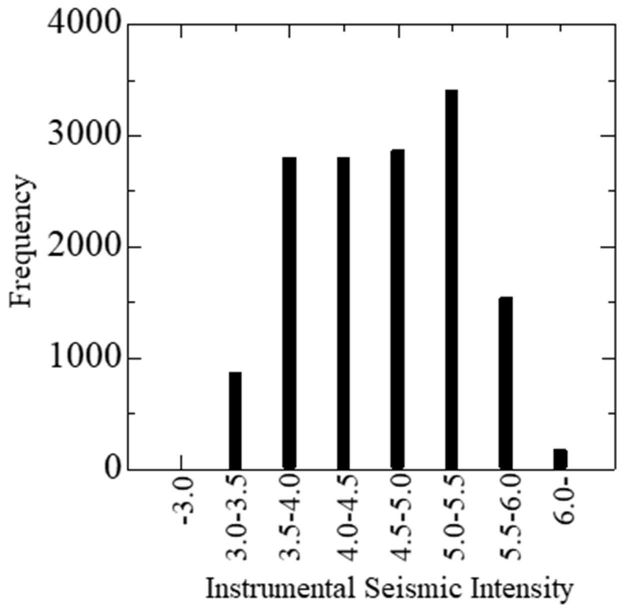

Fig. C.1. Frequency distribution of instrumental seismic intensity at the transmission tower.

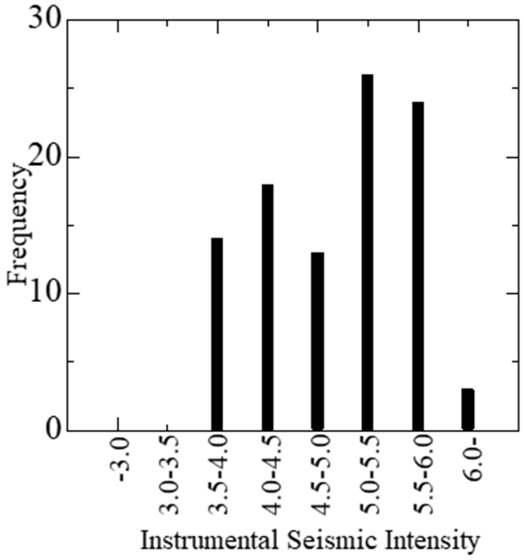

Fig. C.2. Frequency distribution of instrumental seismic intensity at the substation.

References

[C1] Tohoku Electric Power Co., Inc. *Press Releases in 2011*. https://www.tohoku-epco.co.jp/pastnews/2011index.html;2011 (in Japanese) [accessed on 5 June 2021].

[C2] Tohoku Electric Power Co., Inc. *Situations after the Great East Japan Earthquake*. https://www.tohoku-epco.co.jp/pastnews/press/__icsFiles/afieldfile/2012/02/29/12022904_skt.pdf#page=8 ;2012 (in Japanese) [accessed on 5 June 2021]..

[C3] Tohoku Electric Power Co., Inc. *Electricity Supply and Demand Results for March in 2011.* https://www.tohoku-epco.co.jp/pastnews/supply/1183219_1068.html;2011 (in Japanese) [accessed on 5 June 2021].

[C4] Tohoku Electric Power Co., Inc. *Electricity Supply and Demand Results for March in 2011.* https://www.tohoku-epco.co.jp/pastnews/supply/1183285_1068.html;2011 (in Japanese) [accessed on 5 June 2021].

[C5] Tohoku Electric Power Co., Inc. *Electricity Supply and Demand Results for April in 2011.* https://www.tohoku-epco.co.jp/pastnews/supply/1183349_1068.html;2011 (in Japanese) [accessed on 5 June 2021].

[C6] Tohoku Electric Power Co., Inc. *Electricity Supply and Demand Results for May in 2011.* https://www.tohoku-epco.co.jp/pastnews/supply/1183426_1068.html;2011 (in Japanese) [accessed on 5 June 2021].

[C7] Tohoku Electric Power Co., Inc. *Electricity Supply and Demand Results for June in 2011.* https://www.tohoku-epco.co.jp/pastnews/supply/1183476_1068.html;2011 (in Japanese) [accessed on 5 June 2021].

[C8] Tohoku Electric Power Co., Inc. *Electricity Supply and Demand Results for July in 2011.* https://www.tohoku-epco.co.jp/pastnews/supply/1183523_1068.html;2011 (in Japanese) [accessed on 5 June 2021].

[C9] Tohoku Electric Power Co., Inc. *Electricity Supply and Demand Results for August in 2011.* https://www.tohoku-epco.co.jp/pastnews/supply/1183586_1068.html;2011 (in Japanese) [accessed on 5 June 2021].

[C10] Tohoku Electric Power Co., Inc. *Information on empty capacity of the grid.* https://nw.tohoku-epco.co.jp/consignment/system/announcement/; (in Japanese) [accessed on 5 June 2021].
