# Supplementary material for: A framework to estimate a long-term power shortage risk following large-scale earthquake and tsunami disasters
Source: PLoS One. 2023 Mar 27;18(3):e0283686. doi: 10.1371/journal.pone.0283686 (PMC10042361; doi:10.1371/journal.pone.0283686)
Supplement: S1 Dataset — (ZIP) [file pone.0283686.s004.zip › data sets/1 read me first.docx]

Explanation of data sets

All the power facilities are converted into network data (node and link). Each node and link have sequential numbers, and power plants and substations are linked with these numbers. The following files are all the data sets used for simulating the result in the paper.

-Basic settings.xlsx: summary of simulation conditions, location of power plants and substations at the network, power plant capacity and other characteristics

-demand.csv: daily peak demand for target regions

-FragilityParameters.csv: parameters for log-normal distribution to probabilistically estimate the damage

-SIforAll Equipment.csv: Spectral Intensity for all the facilities (power plants & transmission towers.)

-TowersatRoute.csv: start node (tower number) and end node (tower number) for transmission routes.

-WS2.csv: transmission route capacity and distance
